# Top unanswered questions in antimicrobial management of necrotizing soft tissue infections

**DOI:** 10.1017/ash.2025.10268

**Published:** 2025-12-29

**Authors:** Hayato Mitaka, Jeannie D. Chan, Erika Bisgaard, John B. Lynch, Chloe Bryson-Cahn

**Affiliations:** 1 Division of Infectious Diseases, Department of Medicine, University of Colorado School of Medicine, Aurora, CO, USA; 2 Division of Allergy & Infectious Diseases, Department of Medicine, University of Washington School of Medicine, Seattle, WA, USA; 3 Department of Pharmacy, Harborview Medical Center, and University of Washington School of Pharmacy, Seattle, WA, USA; 4 Department of Surgery, University of Washington School of Medicine, Seattle, WA, USA

## Abstract

Necrotizing soft tissue infections (NSTIs) are life-threatening conditions that require prompt surgical and antimicrobial intervention. An upward global trend in invasive group A streptococcal infections, concerning for a synchronous rise in NSTIs, warrants a standardized approach to antibiotic management of NSTIs to optimize care. Emerging data support a shorter antibiotic course following definitive surgical debridement, even in cases with concurrent streptococcal bacteremia. Individualized antibiotic management guided by surgical source control, as opposed to fixed durations, may help minimize unnecessary antibiotic exposure and the resultant adverse events and antimicrobial resistance. The use of clindamycin as an adjunctive anti-toxin antibiotic remains a common practice, though rising resistance and comparative studies suggest linezolid may be a safe alternative. This review aims to offer strategies to optimize antibiotic care in NSTIs by reviewing the growing body of evidence on antibiotic duration, de-escalation strategies, and adjunctive anti-toxin therapy.

## Introduction

Necrotizing soft tissue infections (NSTIs) are rare but life-threatening infections that involve soft tissue from the dermis and subcutaneous tissue to fascia and muscle,^1^ and warrant early recognition and require a combination of prompt surgical debridement and antimicrobial therapy. Group A Streptococcus (GAS) is one of the most implicated pathogens in NSTIs, and the rise of invasive GAS infections worldwide is a concerning trend.^2,3^ Despite high morbidity and mortality associated with NSTIs, there is substantial variation in care patterns and antimicrobial management even at institutions with expertise in NSTIs, since consensus guidelines are largely based on expert opinions in the absence of high-quality evidence.^4,5^ A growing body of literature in NSTI antimicrobial management has emerged. We aim to address the choice, duration, de-escalation strategies of antibiotic treatment and adjunct anti-toxin therapy with a critical appraisal of the literature and offer our viewpoints where there is uncertainty.

## What empiric antibiotic regimen is recommended for NSTI?

**Figure 1.**
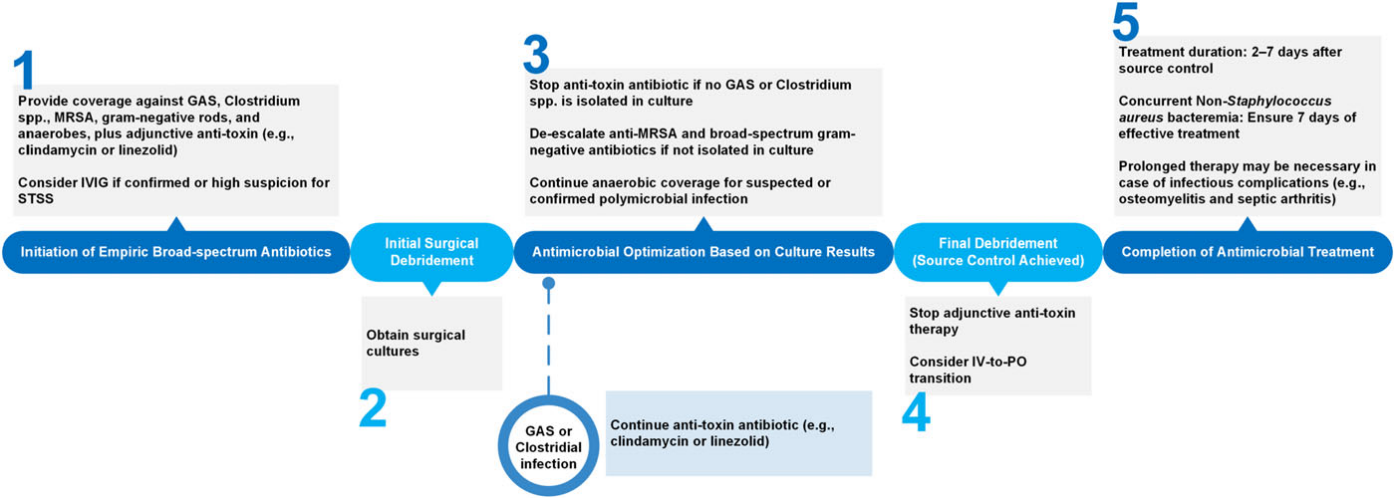
Antimicrobial decision-making points in the management of necrotizing soft tissue infections. GAS, Group A Streptococcus; MRSA, methicillin-resistant Staphylococcus aureus; IVIG, intravenous immunoglobulin; STSS, streptococcal toxic shock syndrome.

Data for empiric antibiotic treatment for NSTI is scarce with guideline recommendations based on expert consensus. The Infectious Diseases Society of America (IDSA) guidelines support empiric therapy with coverage against gram-positive, including methicillin-resistant *Staphylococcus aureus* (MRSA), GAS, and *Clostridium* spp., gram-negative, and anaerobic organisms.^5^ Typically, this includes an intravenous (IV) β-lactam/β-lactamase inhibitor (or a carbapenem) in combination with linezolid or vancomycin plus clindamycin (Figure 1). Although it is often empirically included in the regimen, the value of routine anti-pseudomonal coverage is unclear as *Pseudomonas aeruginosa* is an uncommon pathogen, representing only 1% of NSTIs.^6^

## What is the optimal duration of antibiotic treatment of NSTIs?

Duration of antibiotic therapy for NSTIs is not well defined due to lack of randomized controlled trials (RCTs). IDSA guidelines recommend continuing antimicrobials until surgical debridement is no longer necessary, along with clinical improvement, including being afebrile for 48–72 hours.^5^ The median duration of antibiotic therapy for NSTIs varied across academic institutions with high NSTI volumes, ranging from 9 to 16 days.^4^ Horn et al sought to evaluate patient characteristics that influence antibiotic decisions to help determine optimal duration of therapy.^6^ Among NSTI patients without a complicating secondary infection, the median overall antibiotic duration was 9.8 days, and 7.0 days after the final debridement. They concluded that a 7-day course after final operative debridement may be sufficient, and clinical characteristics such as leukocytosis and fever were not associated with failure of antibiotic discontinuation.

In recent years, accumulating evidence has supported shorter courses of treatment for most bacterial infections, and the increasing focus on patient safety has led to efforts to curtail unnecessary antibiotic use. Several observational studies have demonstrated that shorter antibiotic therapy for NSTIs is safe and effective without negatively affecting patient outcomes. Kenneally et al reported no significant differences in recurrence (1.4% vs 3.6%; *P* = .697), mortality (1.4% vs 4.4%; *P* = .476), or ICU length of stay (1 vs 2 days; *P* = .300) between patients treated with ≤48 hours of antibiotics after surgical debridement compared to those treated with longer courses,^7^ while overall hospital length of stay was shorter (7 vs 10 days; *P* = .011) in patients who received the abbreviated course. Similarly, Terzian et al conducted a prospective study of 151 patients with NSTI following an institutional guideline change that recommended stopping antibiotics after 48 hours following source control. The median duration of antibiotic therapy was 180 versus 48 hours in the pre-and postimplementation groups, respectively, and there was no significant difference in treatment failure rates (5.9% vs 6.3%; *P* = .93) or 30-day mortality (6.7% vs 6.3%; *P* = .94).^8^ Furthermore, a systematic review and meta-analysis of observational studies comparing shorter (≤7 days) versus longer (>7 days) antibiotic duration for NSTIs, which included 532 patients from four studies, demonstrated no significant difference in mortality rates, recurrence rates, limb amputation, or *Clostridioides difficile* infection (CDI) rates between shorter and longer duration.^9^ These observational studies, however, did not adequately adjust for clinical covariates or account for other complications such as concurrent bacteremia, septic arthritis, and osteomyelitis. There is an ongoing pilot RCT evaluating the safety of a 48-hour versus a 7-day course of antibiotics after source control,^10^ which will inform clinicians in determining the shortest effective antibiotic duration for NSTIs.

Fournier’s gangrene, an NSTI of the perineum, is often polymicrobial involving enteric gram-negative bacteria and anaerobes. It is postulated that Fournier’s gangrene is unique from non-perineal NSTI with lower mortality and a potentially less aggressive natural history.^6^ Recent observational studies suggest that Fournier’s gangrene may be treated with an even shorter course of antibiotic treatment compared to non-perineal NSTIs. In the NSTI registry study at a quaternary referral center, which included 165 (37%) patients with Fournier’s gangrene, the overall antibiotic duration was shorter in patients with perineal involvement compared with those with non-perineal NSTIs (8.3 days vs 10.6 days).^6^ Another single-center, retrospective study of 168 patients with Fournier’s gangrene also showed no significant difference in mortality, successful primary closure, surgical site infection, or CDI among those treated with a shorter (≤7 days) versus longer course of antibiotic therapy, and there were no cases of recurrent Fournier’s gangrene in any antibiotic duration stratification.^11^

Similar to antimicrobial treatment for uncomplicated skin and soft tissue infections (SSTI), existing observational studies consistently support 7 days or fewer of antibiotics for NSTIs, provided that source control has been achieved (Table 1). This approach also aligns with the 2020 Surgical Infection Society Guidelines, which endorse a shorter course of antibiotic treatment (<7 days).^12^ In the absence of compelling benefits from a prolonged course of antibiotics, clinicians should recognize that each additional day of antibiotic exposure is associated with an increased risk of adverse events^13^ and antibiotic resistance.^14^

**Table 1.** Studies evaluating antibiotic treatment duration for necrotizing soft tissue infections^6–8,11,52^

Author	Year	Design	Population	Treatment duration		Outcomes	Conclusion
(Country)				Shorter	Longer		
Lauerman^11^ (USA)	2017	Retrospective cohort; single center	168 patients with Fournier gangrene	≤7 days (N = 28)	>7 days (N = 140)	Mortality, CDI, recurrence, SSI	No significant difference in mortality, primary closure, SSI, or CDI across the treatment durations. No cases of recurrent Fournier gangrene.
Valadez^52^ (USA)	2021	Retrospective cohort; two affiliated centers	142 patients; mixed NSTI types	≤7 days (N = 49)	>7 days (N = 93)	Mortality, amputation, CDI, 30-day readmission, length of stay	No significant difference in mortality, amputation, CDI, length of stay, or 30-day readmission.
Kenneally^7^ (USA)	2022	Retrospective cohort; single center	322 patients; mixed NSTI types	≤48 hours after final debridement (N = 71)	>48 hours after final debridement (N = 251)	Mortality, recurrence, length of stay, clinical success rate	Shorter group was associated with shorter hospital length of stay (7 vs 10 days; *P* = .011). No difference in recurrence (1.4% vs 3.6%; *P* = .697), mortality (1.4% vs 4.4%; *P* = .476), clinical success (47% vs 45%; *P* = .893), or ICU length of stay (1 vs 2 days; *P* = .300).
Terzian^8^ (USA)	2022	Prospective cohort; single center	151 patients pre and postinstitutional protocol change where antibiotics are stopped within 48 hours of source control	Postintervention (N = 32) Median duration: 48 hours (IQR, 32.3–100.8)	Preintervention (N = 119) Median duration: 180 hours (IQR, 100.7–318.8)	Treatment failure, mortality	No significant difference in treatment failure (6.3% vs 5.9%; *P* = .93) and mortality (6.7% vs 6.3%; *P* = .94).
Horn^6^ (USA)	2023	Prospective registry analysis; single center	441 patients (360 with uncomplicated NSTI)	No comparison	No comparison	Mortality, length of stay, amputation, CDI	CDI risk increased with longer duration. Among those without a complicating infection, the median duration of antibiotic administration was 9.8 days overall, and 7.0 days after the final debridement.

NSTI, necrotizing soft tissue infection; CDI, *Clostridioides difficile* infection; SSI, surgical site infection; ICU, intensive care unit; IQR, interquartile range.

## How should “day 1” of antibiotic treatment for NSTIs be determined?

Many patients with NSTI are transferred from smaller hospitals to referral centers after receiving initial IV antibiotics with or without initial debridement,^4,6^ creating uncertainty in designating a clear “day 1” of treatment when assessing total antibiotic duration. Given that surgical debridement is the cornerstone of NSTI management, continuation of antibiotics should be guided by surgical findings of infected and surrounding necrotic tissue and/or bacterial burden.^7–9^ We advocate that surgical source control be considered achieved at the time of the final major debridement when no further procedures are planned that would significantly increase the size of the operative wound in any dimension. Although patients often return to the operating room for subsequent “clean-up” procedures, if no significant necrosis or residual infection is identified, these should not be considered additional major debridement and therefore should not change day 1 of antibiotic when source control was deemed achieved. We propose that antibiotic duration should be determined relative to the date of source control as “day 1” rather than a fixed duration, which helps reduce unnecessary antibiotic exposure.^7–9^

## Is a longer course of antibiotic treatment warranted for concurrent streptococcal bacteremia?

Up to 10%–15% of NSTIs are complicated by bloodstream infections,^4,6,8^ which can occur through either severe soft tissue infection leading to secondary bacteremia or seeding of deep tissues via bacteremia. Timely source control is the cornerstone of the management of NSTI-associated streptococcal bacteremia; thus, persistent bacteremia remains exceedingly rare once the focus of infection has been debrided.^15^ Uncomplicated bacteremia, while not precisely defined, generally implies rapid clearance of bacteremia in the absence of a difficult-to-eradicate infectious source and concerns for endovascular or metastatic infections.^16^ Unlike *Staphylococcus aureus*, which has a high propensity for establishing satellite foci of infections that are difficult to obliterate, streptococci are a diverse group of bacteria that can cause a wide spectrum of infections, most notably SSTIs. Although streptococci may be implicated in infective endocarditis, the risk appears to be species-dependent, and the prevalence of *S. pyogenes* attributed to endocarditis is extremely low, estimated to be 1.9%.^17^ Among patients in an NSTI registry study over a four-year period, only one out of 441 reported having endocarditis.^6^ Although the optimal treatment duration for uncomplicated streptococcal bacteremia remains undefined, a common clinical practice has been defaulted as 14 days.^16,18^ Despite the lack of consensus guidelines addressing optimal duration in uncomplicated streptococcal bacteremia, observational data supporting shorter courses are accumulating. Several retrospective studies across multiple healthcare systems have consistently demonstrated that shorter therapy (median duration ranging from 7 to 10 days) is safe and effective compared to longer duration, with no significant differences in recurrence of bacteremia, readmission, or mortality.^19–22^ Specifically, a retrospective study of 286 patients with *S. pyogenes* bacteremia in Australia, where the predominate source of infection was skin and soft tissue in >70% of patients, found no significant difference in 90-day mortality between patients who received ≤10 days of antibiotic therapy and those who received longer courses (1.5 vs 1.7%; *P* = 1.0).^22^ Moreover, the BALANCE trial, the largest RCT comparing 7 versus 14 days of therapy among 3,608 patients with non-*Staphylococcus aureus* bacteremia, comprising 55% of critically ill in the ICU, demonstrated non-inferiority of the shorter duration, which was consistent in the subgroup of 187 patients with SSTI.^18^

Streptococcal bacteremia secondary to NSTIs should not be an exception unless there remains an uncontrolled source that cannot be surgically managed in which “day 1” of antibiotics should be reset to the day of source control. It is reasonable to discontinue antibiotics for streptococcal bacteremia after a total duration of 7 days as long as source control has been achieved for at least 48 hours.

## What is the adjunctive anti-toxin antibiotic of choice? How long should anti-toxin antibiotics be continued? What is the risk of serotonin toxicity associated with linezolid as anti-toxin therapy?

The IDSA guidelines recommend the combination of penicillin and clindamycin for NSTIs caused by GAS^23,24^ or clostridial gas gangrene^25,26^ based on the anti-toxin effects of clindamycin, despite the lack of high-quality RCTs.^5^ Notably, the addition of clindamycin has been demonstrated to improve mortality in invasive GAS infections among observational studies.^27,28^ However, the rise in clindamycin resistance among GAS has called into question the role of clindamycin as the adjunct protein synthesis inhibitor of choice, including a study where clindamycin-resistant beta-hemolytic streptococcal NSTI was associated with increased odds of limb amputation.^29^ The pros and cons of linezolid as its replacement were nicely summarized by Cortés-Penfield and Ryder.^30^ Most recently, a large-scale retrospective cohort study with target trial emulation attempted to assess the efficacy of adjunctive linezolid compared to clindamycin in hospitalized patients with invasive GAS infections using administrative billing data from US hospitals.^31^ Among 1,095 patients with invasive GAS who received β-lactams; the majority (56%) had bacteremia, while NSTI (n = 40, 3.7%) and streptococcal toxic shock syndrome (STSS) (n = 81, 7.4%) were reported sources. Among clindamycin-treated (n = 829) and linezolid-treated (n = 266) patients, the average duration of adjunctive therapy was 4.9 days and 4.5 days, respectively. There was no significant difference in in-hospital mortality (7% vs 9.8%; adjusted risk ratio, 0.92 [95% confidence interval (CI), 0.42–1.43]), median length of stay, or CDI between the two groups. Given the limited feasibility to conduct an adequately powered comparative RCT, this is the best available study data to-date, affirming linezolid as an acceptable first-line adjunctive therapy. Furthermore, the advantage of linezolid over clindamycin is its reliable activity against MRSA, eliminating the need for empiric vancomycin. Some academic institutions have already replaced clindamycin and vancomycin with linezolid^32^ and have demonstrated reduced antibiotic-related adverse events, including CDI and acute kidney injury.^33^

The optimal duration of adjunctive anti-toxin antibiotics, however, remains speculative once the patient is hemodynamically stable and the bacterial burden has been sufficiently reduced by surgical debridement. The additional benefits of continuing anti-toxin therapy are unclear, while antibiotic-associated adverse reactions are highly prevalent and costly to our health systems.^13^ CDI is commonly linked to antibiotic-associated disruptive changes to the intestinal microbiota. Specifically, clindamycin is associated with the highest risk of CDI among all antibiotics.^34^ Recent observational studies have reported that approximately 4% to 6.5% of patients with NSTI treated with clindamycin developed CDI^6,31,32,35^; furthermore, each additional day of clindamycin exposure was associated with a 6% greater odds of CDI.^6^ We propose that the duration of anti-toxin antibiotic should be guided by microbiological evaluation. If blood and surgical cultures do not grow GAS or clostridial species, adjunctive anti-toxin antibiotic should be discontinued, along with other broad-spectrum empiric antibiotics which should be optimized based on culture results. As anaerobes may be difficult to cultivate resulting in late growth, it is reasonable to continue anaerobic coverage in polymicrobial infection such as Fournier’s gangrene. For patients with NSTI caused by GAS or *Clostridium* spp., it is reasonable to continue adjunctive anti-toxin antibiotics until the patient has clinically improved and achieved surgical source control.

Linezolid is a weak, reversible monoamine oxidase inhibitor and may increase the risk of serotonin toxicity when co-administered with serotonergic agents including selective serotonin reuptake inhibitors (SSRIs), psychotropic agents, and opioids.^36^ The average onset to serotonin toxicity from linezolid administration was 46 ± 30 hours, ranging from 1 hour to 20 days.^36^ Symptom resolution was achieved within 24–48 hours after discontinuation of the offending agent(s) in over 75% of cases. Despite FDA warning, evidence from hospitalized populations suggests that serotonin toxicity is uncommon and generally reversible. A pooled analysis of 20 clinical trials found a 0.5% incidence of serotonin toxicity in patients receiving linezolid with a serotonergic agent.^37^ Observational studies in inpatient settings have shown even lower rates—<0.01% in one analysis of 1,170 patients.^36,38^ Even in ICU settings, where opioids are routinely administered for sedation and pain control in NSTI patients, the risk remains extremely low (1.7%).^39–41^ Given the reversibility of serotonin toxicity, linezolid may be safely co-administered with other serotonergic agents or opioids with clinical caution and close monitoring.

## What is the role of intravenous immunoglobulin (IVIG)?

STSS, characterized by hypotension and end-organ failure, is a life-threatening complication of NSTI. Although evidence supporting the benefits of intravenous immunoglobulin (IVIG) is conflicting, IVIG has been proposed as an adjunctive therapy to enhance bacterial opsonization and toxin neutralization, particularly when GAS is implicated.

The only randomized placebo-controlled trial designed to evaluate the efficacy of IVIG in STSS was terminated early due to low recruitment after enrolling 21 patients.^42^ Despite the lack of statistical power to detect a difference in mortality, there was a trend toward improved SOFA scores and survival among the 10 patients randomized to IVIG. Several observational studies have since evaluated the impact of IVIG but have yielded inconsistent results.^43–45^ The inherent risk of bias, severity of illness, and the inconsistent co-administration of clindamycin further complicate the interpretation of retrospective data. Parks et al conducted a meta-analysis of 165 clindamycin-treated patients with STSS (70 IVIG and 95 non-IVIG patients) and reported that administration of IVIG was associated with a reduction in mortality from 33.7% to 15.7% (risk ratio 0.46 [95% CI, 0.26–0.83]).^43^ Notably, the largest retrospective cohort study examining the effect of IVIG in vasopressor-dependent NSTI patients was excluded from this meta-analysis.^44^ In that study, no significant differences in hospital length of stay or mortality were observed between 161 IVIG-treated patients and 161 non-IVIG-treated patients who were propensity-score matched. However, when the subset of 49 clindamycin-treated GAS patients was incorporated into the meta-analysis, the finding corroborated the mortality benefit of IVIG (RR 0.51 [95% CI, 0.29–0.90]). Furthermore, a multi-center prospective study also identified the absence of IVIG administration as an independent risk factor for 90-day mortality among 125 patients with GAS NSTI (97% received clindamycin), although longer wait time to surgery among non-IVIG patients may have biased the outcome.^45^

In contrast, the INSTINCT trial randomized clindamycin-treated NSTI patients to receive either IVIG (25 g/day) or placebo and found no statistically significant differences in self-reported physical function at 180 days, resolution of shock, or mortality, although GAS accounted for only 18% of the study population.^46^ Similarly, a recent cohort study of clindamycin-treated NSTI patients reported no difference in ICU length of stay or mortality between patients with or without IVIG.^47^

Currently, there is insufficient evidence to support the routine use of IVIG in the absence of adequately powered RCTs. Nevertheless, given the high morbidity and mortality associated with STSS, IVIG should be considered in patients with suspected or confirmed STSS. The optimal dose of IVIG remains undefined, and clinicians should be aware of the considerable batch-to-batch variability of polyspecific IVIG preparations regarding the quantity of neutralizing antibodies. One study suggested that a 25 g IVIG is sufficient to achieve plasma neutralizing activity against streptococcal superantigens.^48^ We recommend IVIG 1 g/kg on day 1, followed by 0.5 g/kg on days 2 and 3 for patients with STSS.^42^

## When should the transition to oral antibiotics be considered?

Empiric broad-spectrum antibiotics for NSTIs should be administered intravenously to maximize antibiotic penetration around the necrotic tissue in the setting of impaired pharmacokinetics (PK) and pharmacodynamics (PD) associated with increased volume of distribution and local tissue necrosis.^1^ Once clinical improvement and stability are achieved, transition from IV to oral therapy should be considered, though it has not been explicitly studied in NSTIs, as patients with NSTI are often excluded from observational studies evaluating IV to oral switch. However, observational studies investigating IV-to-PO transitions in uncomplicated streptococcal bacteremia have uniformly reported that this approach was safe and often associated with significantly shorter lengths of stay without negatively affecting clinical outcomes.^49^

Once source control is achieved, NSTI should be viewed no differently than “uncomplicated” SSTI. Thus, transition to oral therapy should be considered once the following criteria are met, as proven in the most challenging infections like endocarditis and osteoarticular infections^50^: (1) the patient is clinically and hemodynamically stable; (2) surgical source control has been achieved; (3) there are no concerns regarding absorption of oral antibiotics from the gastrointestinal tract; (4) a reliable oral antibiotic regimen, supported by published clinical data and/or PK-PD profile for SSTI. Although the major stewardship emphasis should be on ensuring the shortest effective duration of antimicrobial therapy after achieving surgical source control, coupled with timely optimization of antibiotic regimen based on surgical culture data, oral transition after source control is another important stewardship opportunity in reducing the risk of potential harms from IV catheters and IV therapy.^51^

## Conclusion

Despite recent advancements in the optimization of antibiotic duration, de-escalation strategies, and IV-to-PO transition in common bacterial infections, significant knowledge gaps persist in the optimal management of NSTIs. Current evidence suggests that shorter course guided by surgical source control may be as effective as longer therapy while reducing the risks of antibiotic adverse events and emergence of resistance. Linezolid is increasingly considered as an alternative adjunctive anti-toxin agent with the growing resistance among GAS to clindamycin. The lack of robust prospective data for the shortest effective duration after final debridement and transition to oral therapy in NSTI patients, highlights the need for further research to standardize the approach and optimize patient outcomes of this rare yet life-threatening infection.
